# Therapeutic Efficiency of Humic Acids in Intoxications

**DOI:** 10.3390/life13040971

**Published:** 2023-04-09

**Authors:** Janka Vašková, Marek Stupák, Martina Vidová Ugurbaş, Daniel Žatko, Ladislav Vaško

**Affiliations:** 1 Department of Medical and Clinical Biochemistry, Faculty of Medicine, Pavol Jozef Šafárik University, 040 11 Košice, Slovakia; janka.vaskova@upjs.sk (J.V.);; 2 Second Department of Surgery, Faculty of Medicine, Pavol Jozef Šafárik University, 040 11 Košice, Slovakia; 3 Imuna Pharm, a.s., Šarišské Michaľany, 082 22 Presov, Slovakia

**Keywords:** antioxidant, adsorption, heavy metal poisoning, humic acids, chelating, mycotoxin poisoning, radical scavenger

## Abstract

Humins, humic and fulvic acids represent molecules with complex structures. These compounds comprising humic substances (HS) exist naturally in soil, brown coal, peat, and water. They are formed during the decomposition and transformation of organic matter (animal and plant remains) and their formation explains several theories. Within their chemical structures, there are numerous phenolic and carboxyl groups and their derivatives that affect their different properties, such as their solubility in water or their absorption of cations or mycotoxins. The manifold chemical structure of HS alters their polyelectrolyte character and thus their chelating efficiency. For many years, HS have been studied due to their detoxification, anti-, and pro-inflammatory or anticancer and antiviral ability. This article summarizes the antioxidant and adsorption properties of humic acids, highlighting their usefulness in intoxications.

## 1. Introduction

In the last decade, the application of preparations based on humic acids (HAs) has greatly expanded, especially in plant and animal production, where they are used as intensification factors. In medicine, they are used for the treatment of various diseases, especially heavy metal poisoning, diseases of the gastrointestinal tract, and various inflammatory and tumor diseases. Humic acids are natural organic compounds resulting from the chemical and biological decomposition of organic matter (especially plants) and the synthetic activity of microorganisms. Their occurrence in nature ranges from trace amounts (standing water, sandy soils, clay) to units of % (medicinal muds, manure, topsoil). They are mostly found in peat (10–40%), lignite (10–30%) and brown coal (up to 40%). The highest occurrence is in oxyhumolites (50–80%). Oxyhumolites are formed under certain conditions (access to air and water) from lignite or brown coal. The structure and composition of humic acids mainly depend on the conditions of formation, on basic plant biomass, and microbial composition (bacteria, fungi, filamentous fungi). The formation of humic substances is a polymerization process, and therefore the size of the molecules is also affected by age. Humic acids have a polyanionic character and bind ions by various mechanisms, both chemical and physical. Compared to inorganic adsorbents (zeolites), their adsorption capacity is seven to ten times higher. The absorption of humic acids in the gastrointestinal tract of animals is very low, less than 0.1%. The Commission for Veterinary Medicines at the EMEA (The European Agency for the Evaluation of Medicinal Products [1]) allowed the oral use of preparations based on humic acids for all, including food animals, indicating a range from 0.05 to 0.07%. The biological role of humic acids is quite well-researched. Due to the large variability of humic acid molecules, which depends on the source (different biological, chemical, physical, and geological conditions of formation) and the method of preparation, not all preparations may be based on free humic acids, oxyhumolite or sodium humate. Although, they are equally effective. In this work, we will describe the antioxidant and adsorptive properties of humic acids detected primarily in the organism.

## 2. Humic Substances (HS) and Theories of Their Formation

Humic substances (HS) is a collective term for organic substances forming humus. They are compounds with a high molecular weight, which together form a brown to a black, hydrophilic, molecularly flexible, polyelectrolytic substance called humus. These substances create large, stable complexes with each other, which are mainly involved in the formation of the structure, porosity of the soil, the ability to retain water in the soil, and also the ion-exchange capacity (chelation of inorganic cations). From the point of view of elemental representation, mainly carbon, oxygen, hydrogen, nitrogen, and sulfur are present in HS, as part of structurally heterogeneous carbon chains (of which approximately 40–50% are aliphatic, the rest are alicyclic, while aromatic chains make up a total of 35–60%). In terms of functional groups, carboxylates, phenolic and non-aromatic hydroxyl groups, oxo- and quinone groups, amino- and imino groups, as well as esters, amides (cyclic and non-cyclic), acetals and ketals, ether and peroxy groups prevail; in terms of classes of structural components, oligo- to polysaccharides, sugar acids, oligo- to polypeptides, residues of fatty acid chains, lignin fragments, condensed aromatics, which often contain heterocyclic cores, appear in HS molecules. HS are notable for their great structural variability, either in molecular size, ranging from 1 kDa to 10 MDa, the relative content of individual elements, functional groups, or in redox, proteolytic, complexation properties, solubility in water (and other solvents) and therefore their content and availability in individual areas of the lithosphere and hydrosphere [2,3]. The origin of this diversity must be sought in the mechanism of the formation of HS.

There are several theories about the formation of HS, while they prefer different sequences of inorganic, organic, and biochemical reactions, they use synthetic (so-called polymerization theories) or decomposition (depolymerization theories) approaches. Formation of HS in the so-called “process of humification” includes concurrence of several assumed mechanisms, while microorganisms present in water or soil play a significant role here, the presence and action of inorganic catalysts in the form of mineral oxides and salts is essential.

The most frequently discussed theories include the ligno–protein theory (discussed by several authors, e.g., [4,5,6,7]), the phenol–protein theory (polyphenol theory-[6], phenol-autooxidation theory [8]) or the concept sugar–amine condensation (often also known as the melanoidin pathway, or the Maillard reaction), which is mainly used to explain the formation of humic substances in the aquatic environment (plant residues involved in their formation do not contain polyphenolic lignin) but is also relevant in the genesis of soil HS.

### 2.1. Precursors of Humic Substances

The question of which precursors humic substances are formed from in the process of humification is important, if not more relevant in view of their biological properties. The most frequently referred to raw material is lignin, a plant polymer that, together with cellulose and hemicellulose represents the main constituents of wood matter.

**Figure 1:**
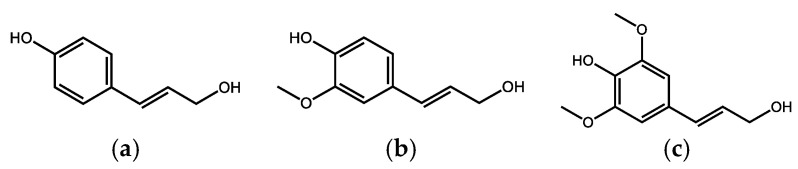
Monomeric phenolic building blocks of lignins: (**a**) *p*-coumaryl alcohol; (**b**) coniferyl alcohol; (**c**) sinapyl alcohol.

Lignin is a polymer derived from a phenylpropenyl structure, containing several phenolic hydroxy groups, together with methoxy groups and a conjugated unsaturated propenyl or hydrated chain, the most common monomers are the so-called monolignols, p-coumaryl alcohol (mainly present in grasses and bamboos), sinapyl alcohol (found in hardwood lignin) and coniferyl alcohol (soft wood lignin) (Figure 1).

These monomers are created by the enzymatic aromatization of carbohydrates and further polymerization known as the lignification process, which creates lignin.

**Figure 2:**
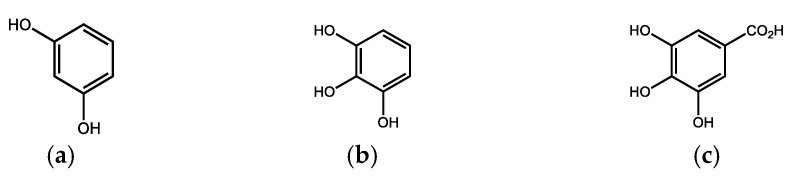
Some phenolic (synthetically important) mycodegradation products of lignins: (**a**) resorcinol; (**b**) pyrogalol; (**c**) gallic acid.

Due to its high chemical resistance and resistance to microbial decomposition, lignin can accumulate in the soil and thus participates to a significant extent in the formation of peat and enters the humification process [9]. However, some basidiomycetes and ascomycetes are capable of degrading lignin, creating the original, abovementioned monomeric phenols, corresponding oxidation products (various phenolic, hydroxyaromatic carboxylic acids, e.g., gallic acid (Figure 2) and their alkylated analogs) and after the subsequent demethylation and decarboxylation to simple phenols (especially resorcinol and pyrogallol, Figure 2) [10].

Several types of fungi (*Aspergillus*, *Epicoccum*, *Hendersonula*, *Penicillium*, *Euratium*, *Stachybotrys*) are able to produce substances similar to humic acids on a nutrient medium containing glucose, sodium nitrate, asparagine or peptone. These dark polymeric substances are composed mainly of phenols, orselinic acid, p-hydroxybenzoic acid, p-hydroxycinnamic acid, anthraquinone units, and melanins [11]. The ectomycorrhizal fungus *Pisolithus tinctorius*, cultured on Melins–Norkrans medium containing sucrose or a mixture of succinic and L-malic acids, was able to produce a brown polymeric substance which, when extracted with NaOH and HCl, behaved similarly to humic acids (HAs) and fulvic acids (FAs) while the IR spectra of individual fractions had features comparable to IR spectra of HAs and FAs. This polymer consisted mainly of various uronic acids, which are considered to be one of the structural “building blocks” of HAs [12]. Other structural units of humic substances include quinones, which are formed by enzymatic (phenolase, laccase) oxidation or autoxidation processes from the abovementioned simple microbial phenolic substances. In addition, the resulting yellow to red colored quinones can simply polymerize to form diquinones, etc. Quinones do not have an aromatic character and are variably chemically modifiable, they readily react with nitrogenous nucleophiles to form imines, oximes, etc. [8].

Although some authors are of the opinion that proteins and amino acids are not components of humic substances and explain their content only by the adsorption of proteins onto HS particles or by their coprecipitation during acid isolation of HS samples, the chemistry of complex proteins rather shows that the nitrogen content in humic substances’ samples (on average 1–5%) can be attributed precisely to covalently bound amino acid or oligo- to polypeptide fragments (to the nitrogen content in HAs molecules, cyclically bound nitrogen atoms in heterocyclic condensed aromatic chains also contribute). Proteins of dead plant and animal organisms and their hydrolysates (amino acids) become the target of saprophytic bacteria and fungi, which enzymatically transform them under both aerobic and anaerobic conditions releasing ammonia. A certain part of the products of protein hydrolysis can become part of the molecules of humic substances through covalent binding to phenolic or quinone cyclic chains [6]. Bound in this way they become resistant to microbial degradation. Some authors attribute the difference in the proteolytic properties of humic and fulvic acids to the content of amino acids—while the FAs molecules contain mainly basic amino acids, the HAs structure contains more amino acids with acidic side chains [13].

Polysaccharides represented in soil organic matter (starch, cellulose, hemicellulose) are considered less available for microbial degradation, in principle, the higher the degree of branching of their chains, the lower their microbial bioavailability. From the point of view of biotic theories of the formation of humic substances, they are rather only adsorbed (dipole–dipole interactions, hydrogen bonds) on the surface of macromolecular particles (or micelles) of humic acids or humins. However, in some structural models of HAs, there are sugar fragments, more specifically ester-bound molecules of alduronic acids, i.e., products of the enzymatic metabolism of monosaccharides [6].

**Figure 3:**
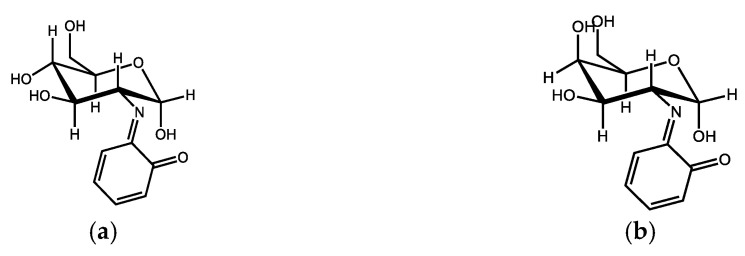
Examples of Schiff base structures derived from aminosaccharides and quinones: (**a**) product of reaction of glucosamine and *o*-chinone; (**b**) product of reaction of galactosamine and *o*-chinone.

**Figure 4:**
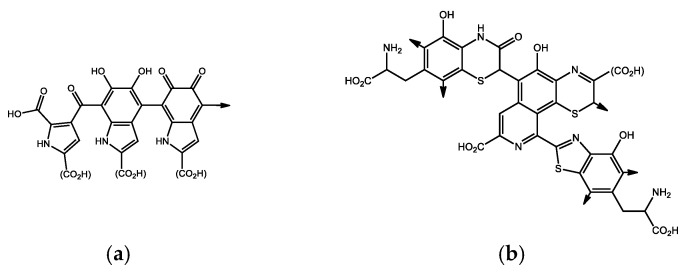
Examples of fragments of polymer structures of melanins: (**a**) eumelanin; (**b**) pheomelanin. Arrows represent possible chain continuation, and functional groups in parenthesis can be replaced by -H or -R.

In terms of the Maillard reaction, it is very likely that carbohydrates enter the process of formation of HS in the form of amino sugars (glucosamine and galactosamine, Figure 3), that at a higher temperature react with phenolics or by the quinonoid parts of the molecules of humic substances, thus creating a basic imine (the nitrogen of which can therefore be found in the heterocyclic parts of the HS molecules). After the reaction with amino acids, basic imines can change to a melanoid aromatic structure, probably to a large extent responsible for the coloring of the molecules in which it occurs and contributes to the radical stabilizing effects of such molecules [8]. From the animal kingdom, we know several variants of melanin (Figure 4) such as eumelanin, resulting from the polymerization of oxidized tyrosine, responsible for the brown and black coloring of the parts of the body in which it is found, pheomelanin (pink to red), also containing benzothiazine nuclei originating from cysteine [14], or neuromelanin, occurring in the black matter of the midbrain in humans—its high chelating affinity towards iron cations and its ability to bind and interact with lipids or various pesticides, e.g., by the neurotoxic herbicide MPP+ (N-methyl-4-phenylpyridinium chloride, cyperquat) leads to conclusions about its neuroprotectiveness [15,16].

The precursors of humic substances can also include other organic compounds present in the soil, namely lipids, nucleic acids, chlorophyll, vitamins, hormones, and recently pesticides and their metabolites, or degradation products, due to their extensive agricultural use [17].

### 2.2. Structure of Humic Substances

#### 2.2.1. Polymer Versus Supramolecular Association Model

In general, HS can be considered as organic heteropolymers, or clusters of low-molecular polyfunctional structures that are held together by relatively strong dipole–dipole interactions, especially hydrogen bonds, hydrophobic interactions (instant dipoles, π–π interactions), or coordination bonds in complexes with metals, and which are preserved (do not break down) under conditions of commonly used techniques for dividing and purifying HS. However, supramolecular associates can be decomposed into HS monomers using weak organic acids (e.g., acetic acid, [18]) or weak inorganic acids (H_3_BO_3_, [19]), which compete for the formation of hydrogen bonds with HS monomers but would not be able to hydrolyze covalent ester or ether bonds. Therefore, the research aimed at verifying the structure of HS molecules cannot yet be considered finished, new scientific works dealing with theoretical software for computational simulations of model molecules and the evaluation of their stability and theoretical properties, as well as practical spectral measurements (especially the measurements of ^1^H and ^13^C NMR samples in solutions and in the solid phase, IR spectra, fluorescence spectra or X-ray analysis), are continuously published, with the intent to reveal a real picture of the structure of HS. Nowadays, it is probably not possible to talk about a kind of unified structural model, although many authors of recent works in this field rely on, and proceed from, models proposed in the second half of the last century. Due to the different source materials for the synthesis of HS, different climatic, chemical, and biological conditions, and different length stages of humification in different localities, it is generally accepted that no two molecules of humic substances are likely to be exactly the same [20].

There are several criteria according to HS can be systematized, the most common being the origin (source) of HS, color, proteolytic properties, molecular size (molecular weight), and content of functional groups. In general, it is most convenient to use different proteolytic properties or molecular sizes for the sorting of humic substances, which has its origin, and is also used in, the procedures for fractionation and purification of HS. According to this, humic substances are divided into three basic fractions: humins, humic acids and fulvic acids. The properties of individual types of humic substances are briefly summarized in [21]. This description can be considered as data for the molecules of individual types of humic substances, correlating with the so-called “polymer model” of the structure of humic substances [4,6,8], or for the properties belonging to the clusters isolated under the given conditions and showing the properties of the given group of humic substances rather consistent with the so-called “supramolecular association model” [22]. The mentioned structural models have several opponents or supporters in the scientific community, who offer arguments for and against (measurement and analysis results), however, knowledge about humification and carbonization processes (dehydration, deoxygenation, and polymerization) offers the idea of partial acceptability of both models, i.e., of coexistence of polymeric molecules of humic substances and supramolecular associates (e.g., micelles) formed by smaller molecules. This hypothesis certainly does not contradict the variability of the published physical–chemical properties or biological activities of humic acids or fulvic acids from different sources and different locations.

#### 2.2.2. Oxidative Polymerization of Humic Substances

Piccolo [22] demonstrated that humic acids can be polymerized in vitro using H_2_O_2_ and horseradish peroxidase (HRP). Moreover, albeit this enzyme is characterized by maximal activity at pH 4.7, polymerization occurred more rapidly at pH 7.0, since due to the reduced possibility to form hydrogen bonds under these conditions, the HS monomer units were more mobile, and the radical course of the polymerization reaction was thus more likely. We can assume that similar reactions can also take place in vivo at physiological pH, e.g., in the mitochondria of animals or humans, where there is usually an environment with increased levels of reactive oxygen particles of a molecular (e.g., hydrogen peroxide) or radical nature (e.g., hydroxyl radical, superoxide radical anion), and where enzymes with peroxidase activity are also found (e.g., glutathione peroxidase, GPx). In this way, e.g., HAs could participate in reducing the levels of some reactive oxygen species (ROS) and antioxidant protection of cells.

#### 2.2.3. Classification of Humic Substances

The name fulvic acids (FAs) refers to their coloring (lat. “fulvus”-yellow). They represent the soluble fraction of HS, both in acidic and alkaline environments, from which their amphoteric character can be concluded. Their solubility in water is mainly due to the presence of ionizable carboxyl or hydroxy functional groups in their molecules capable of forming hydrogen bonds with polar solvent molecules. The larger number of oxygen-containing functional groups in comparison to HAs is also reflected in the relative representation of carbon and oxygen. Their molecular weight ranges in the order of thousands of g/mol (Da). Depending on the theory of HS formation, they are either products of enzymatic cleavage of HAs or act as their precursors.

In terms of humic carbon content, humic acids represent the largest fraction of humic substances in nature, HAs make up approximately 60–80% of soil HS. They represent an intermediate stage between FAs and humin; since as mentioned above, they are either a product of the reduction and polymerization of FAs or oxidation and degradation of humin. HAs have a lower content of carboxyl groups than FAs, but more than humins. In HAs compared to FAs, quinone oxo groups predominate over aliphatic ones, and skeletal chains are more aromatized, although less than in humin molecules.

HAs compared to FAs also have a greater O/C ratio—while it is at the level of 0.76 in FAs, it is only 0.5 in HAs [23]. The HAs’ color (brown, gray to black material) reflects a higher degree of electron delocalization and polymerization, although some papers (e.g., [18]) discussed a supramolecular association of smaller molecules held together mainly due to strong dipole–dipole interactions (especially hydrogen bonds). Hosse and Wilkinson [24] described the di- to trimerization of HAs in solutions with low pH. In Sparks [25] the predicted structure of up to a hexameric cluster was published. According to the properties of HAs isolated by acid–base extraction, the molecular weight of such (or larger) clusters (or polymer molecules) ranges from 2–200 kDa [21], but according to Havel et al. [19] HAs’ monomers are relatively small molecules with molecular weights of 100–860 Da. One of the possible structures was proposed by Stevenson [6], this one contains a covalently bound peptide chain and a covalently bound alduronic acid, another, rather polymeric model, was proposed by Schulten and Schnitzer [26].

Humins represent a fraction of humus substances that cannot be extracted from soil or lignite material either by acidic or alkaline solutions and therefore form an insoluble residue after FAs and HAs extraction. It is a generally black material, of low polarity, containing the fewest polar functional groups. Some authors [27] state the absence of carboxylates and phenolates, oxygen is represented mainly in carbonyl groups. Humin molecules have a polycondensed aromatic character, while they also contain heteroatoms in the rings. Thus, humus represents a fraction of organic matter in a more advanced stage of humification, further reduction in oxygen content, and aromatization under increased pressure and temperature essentially represent the carbonization process.

## 3. Biological Properties of Humic Substances, Mainly Humic Acids

The therapeutic use of humic acids in the form of peat origin dates back to the first half of the 19th century, when peat baths became popular in the treatment of various skin and rheumatic diseases. Due to its excellent adsorption properties, it was used as a dressing material. In the middle of the 20th century, the effects of peat for external use were already very well documented. Many studies of this period are summarized by Hildebrandt [28] in his review. Ideas for the internal use of peat preparations were developed in the first half of the last century. For the first time, peat extracts were administered internally by pediatricians in Poznań in 1950 [29]. Research in the field of internal use of peat extracts developed mainly in Russia and Poland and led to the patenting of the drugs Torfot (Russia) and Tolpa Peat Preparation (TPP, Poland). These preparations have been valued for their antibacterial, antitoxic, antiradical, as well as immunomodulating, and anti-inflammatory properties [30]. However, works from the last period point to significant shortcomings in the study of the properties of TPP and indirectly recommend only its external use (summarized in the review by Drobnik and Stebel [31]).

Humic acids have a lower ion exchange capacity than fulvic acids, but they are nevertheless capable chelators of metal cations. Thanks to this property, HAs can be used in waste management as a filter, capturing heavy metals, or as a means of removing environmental pollution by such metals [32]. In addition to heterolytic dissociation, carboxyl, and phenolic, and polyphenolic hydroxyl functional groups can also release a hydrogen radical when reacting with reactive particles (e.g., ROS) and thus create more stable, delocalized, non-reacting phenoxy-, carboxy- or phenyl radicals (or other) thus participating in their antioxidant properties [33].

In the study of their properties on the animal organism an important finding was their low toxicity [34], and no side effects even after long-term use (91 days) [35]. However, HAs represent a large heterogeneous group of structures, the specific properties of which may vary slightly depending e.g., on the origin, natural occurrence, or extraction procedure. Complex weight-of-evidence toxicological evaluation of HAs and FAs preparation derived from lignite showed a lack of genotoxic potential and no organ or general toxicity in 90 days oral administration to rats [36].

### 3.1. Antioxidant Properties of Humic Acids

#### 3.1.1. Radical Scavenging Activities of Humic Acids

Several authors have published the antioxidant properties of HAs [27,37] showing protective functions, disruption of the radical chain reaction and thus the capability to prevent damage to cell membranes and biological macromolecules. In the reaction with the hydroxyl radical and ozone, a positive correlation was found between the antioxidant activity of HS and the concentration and amount of unsaturated C = C bonds in their structure. A statistically significant relationship between the rate of oxidation and the C/H ratio (an indicator of the degree of structural unsaturation) was observed. The radical scavenging effect was manifested at high concentrations of HS, and the antioxidant activity was dependent on their structure [38]. Khiľko et al. [39] evaluated the antioxidant capacity of lignite-derived HAs by comparing the ability to inhibit the radical chain reaction of cumene and ethylbenzene initiated by azobisisobutyronitrile (AIBN) and dimethylsulfoxide (DMSO). The authors concluded that the rate of oxygen absorption was significantly reduced in the presence of HA, and at higher concentrations (10 g·L^−1^) the oxidation process was completely stopped. FAs show antioxidant activity, depending on their concentration, but are somewhat less effective than the mentioned reference compounds [40]. Radical-chain-initiated oxidation of 1,4-dioxane (I) under standard conditions was used to assess antioxidant activity by Avvakumova et al. [41]. In an in vitro experiment using a hydrocarbon model [42], they observed that in the presence of HS, the oxidation of this substrate is inhibited, while this effect increases with increasing concentration. Antioxidant properties in hydrocarbons’ oxidation and a pro-oxidant effect in vitamin C oxidation were shown by Smirnova et al. [43]. Measuring the antioxidant capacity of commercially available samples of HAs, Nikolaev et al. [44] found an increase in antioxidant capacity between pH 3.75 to 6.80. The 2,20-azinobis-(3-ethylbenzothiazoline-6-sulfonic acid cation radical assay also by Klein [45], the 2,2-diphenyl-1-picrylhydrazyl (DPPH) assay by Zykova et al. [46], and anthraquinone-2,6-disulfonate (AQDS) by [47] were used. HAs from a natural source in Hungary were capable of scavenging O_2_^−^ up to 20% and −OH over 80% [48]. The antioxidant capacity of HAs by reduction of the complex of ferric ions (Fe^3+^)^−^ was determined by Tarasova et al. [49], as well by Cu (II) reduction to Cu (I) assay in the study of Karadirek et al. [50]. A strong correlation between total phenolic content and antioxidant activity of HS was found [51]. Recently, the study of Klein et al. [52] demonstrated that phenolic as well as non-phenolic moieties of HAs (e.g., carbohydrates) provide antioxidant activity. The physicochemical and radical scavenging properties are affected by the method of extraction [53]. It can be assumed that these remarkable properties of humic acids are the basis of the antioxidant effects in organisms.

#### 3.1.2. In Vitro and In Vivo Efficacy of Humic Acids

Mitochondria are the main metabolic location for the unregulated generation of reactive oxygen species (ROS), where O_2_^−^ is mostly generated during the process of oxidative phosphorylation. Although many mitochondrial proteins can be manipulated to produce O_2_^·−^, the physiological relevance is mostly limited. Therefore, either some in vitro or in vivo experiments with HAs at the recommended prophylactic dosage of 0.1% and under the influence of certain compounds and intoxications were tested. There is a significant decrease in superoxide dismutase (SOD) activities after HA treatment irrespective of being dissolved in DMSO or direct addition to a rat liver mitochondria suspension. This finding in itself would not be so dramatic; however, it is known that there is a threshold of intramitochondrial O_2_^·−^ formation before scavenging by O_2_^−^ drops and peroxidases are exceeded, and sufficient H_2_O_2_ is generated to be measured externally [54,55]. A corollary is that there may be significant O_2_^·−^ formation within mitochondria that goes undetected, which is supported by the finding that H_2_O_2_ efflux from mitochondria increases with peroxidase inhibition [54,56]. There are two main modes of operation by isolated mitochondria that lead to extensive H_2_O_2_ efflux; one is a highly reduced coenzyme Q (CoQ) pool [57]. Some similarities between CoQ and the HA molecular structure supposed that it could interfere with the mitochondrial redox system. The presence of numerous structures similar to quinones in HAs predetermines their role in overdrawing electron acceptance readily donated within the HA molecule without the involvement of mitochondrial enzymes reducing hydrogen peroxides. Aeschbacher et al. [58] evaluated that the presence of electron acceptors (quinone) and electron donors (phenol) depends on the origin of HAs. It also confirmed the hypothesis that phenolic groups in HAs slow down the oxidative transformation of quinones. No significant change was observed in the activity of glutathione peroxidase (GPx). In addition to the lack of changes in synergistically working glutathione reductase (GR), the level of reduced glutathione (GSH) pointed out that the electrophilic properties of HAs markedly balance the mitochondrial redox status [48]. When the level of mitochondrial respiration was measured, it was found that ADP-limited respiration showed an elevation in state 4, when humic acids were present. Additionally, the maximal activity of the respiratory chain, in state 3, showed an identical tendency to increase. An increase in state 4 may indicate lowered coupling by proton gradient dissipation. This partial uncoupling effect alone could prevent ROS formation and increased phosphorylation thus preventing the mitochondrial electron transport chain inhibition [59].

Evaluation of antioxidant properties of HAs in in vivo experiments seems to be more complicated. The concentration of the enzyme responsible for O_2_^−^ production will vary with organism, tissue, state, age, or hormonal status, and may underlie many of the changes in ROS production capacity between tissues [60]. For example, complex I content may explain the different maximum capacities of mitochondria between the species [61]. However, these in vivo measured parameters may also have another cause for the differences, since mitochondrial H_2_O_2_-degradation may vary considerably with the condition and history of the organelle [62]. In a number of previous experiments carried out on chickens, it was found that the beneficial effect was observed after 3 weeks. The results of in vivo experiments under experimental, and the other within a poultry fattening farm, on isolated liver mitochondria [63,64] are much the same as those found in vitro regardless of the differences between the representatives of the classes of vertebrates. One aspect to consider is the fact that, once taken up, HS are able to migrate to organs or organelles and may provoke stress response reactions [65]. They have both non-specific and specific effects. The non-specific effects are physical and chemical membrane irritation, induction and modulation of biotransformation activity, induction of chemical defense proteins, the development of internal oxidative stress, and the induction of ROS defense enzymes. All organisms have the means to rid themselves of chemical burdens (exotic food chemicals, xenobiotics, etc.), i.e., they have developed so-called biotransformation pathways. HS also behave like chemical clues in the biotransformation pathway. Since HS possess a variety of functional groups, we assume that the Phase II enzymes of the biotransformation system (conjugation reactions with glutathione), are subject to modulation upon HS exposure [65,66]. Considering the interdependencies between the activities of enzymes and levels of GSH in comparison between the three organs (the liver, kidney, and plasma), the results are favorable. The redox potential of GSH is not lost in either kidney or circulating plasma, demonstrating the antioxidant effect of HAs [64].

In a study dealing with the use of HAs in Persian, Arabic, Chinese, and Indian traditional medicine [67,68], the anti-cancer effect of HAs is described, mainly due to the antioxidant protection of cellular components, especially DNA, against the harmful oxidative influence of superoxide anion or hydroxyl radical. Likewise, the administration of HAs can be useful as a supportive treatment alongside cancer chemotherapy, where it protects tissue cells around the application of chemotherapeutics and prevents (mitigates) oxidative damage to these cells. Antioxidant effects of HAs can be used as an anti-aging therapy; they reduce the risk of oxidative damage to cells and thus slow down the aging process. As a consequence, vitality and improvement of the overall health of the organism are observed in users [69].

The antioxidant properties of HS can be used in wound healing. Wound healing requires oxygen, and ROS are generated in the process of phagocytosis. In the presence of molecular oxygen, semiquinones produce O_2_^−^, which is converted to hydrogen peroxide in the presence of superoxide dismutase. Hyperoxide ions produce −OH by reaction with transition metals in the Fenton reaction or hydrogen peroxide in the Harber–Weiss reaction [70]. In an animal study, Ji et al. [71] confirmed the ability of sodium humate to promote healing. Wound healing by sodium humate is probably mediated through the transforming growth factor (TGF-β) signaling pathway. Higher content of hydroxyproline and increased wound contraction were also found in the tissue of rats during healing. Overall complex positive effects of HAs were shown on the condition of the dentition and the oral cavity. HAs prevented alveolar bone loss, reduced inflammation [72], showed antimicrobial activity [73], and enhanced healing in the oral cavity in rats [74].

An in vitro study by Ho et al. [75] showed the capability of HAs to reduce Fe^3+^ to Fe^2+^, and reduce and release iron from ferritin storage. Although this in vitro finding is considered a mechanism of HAs iron toxicity, in vivo effects of HAs were predominantly positive. The protective effect of HA against iron-induced hepatotoxicity and cardiotoxicity through antioxidant activity at the biochemical and histological level was confirmed by Cagin et al. [76]. This effect is manifested by the reduction of ROS, the inhibition of lipid peroxidation, the strengthening of the enzymatic and non-enzymatic antioxidant systems and can be based on the ion-exchange properties of HAs. The hydrated bis-dibenzo-pyrone ferrate complex structures in HS prevent Fenton or Haber–Weiss reaction radical formation, but in phagocytic cells facilitates immunological response by releasing free radicals with microbicidal activities. HS allows conformation and morphological changes within cells, and selective oxidoreductase activities [77]. Other anti-inflammatory effects were attributed to the ability to decrease in pro-inflammatory cytokines and acute phase protein after HAs’ preparation application [78,79,80]. Khuda et al. [81] found the HS anti-inflammatory effect is boosted by cyclooxygenase and lipoxygenase inhibition in a dose-dependent manner, and possibly other vasoactive compounds, like serotonin and histamine inhibition. HS and its different fractions have shown efficiency in wide spectrum antiviral and immunostimulating activities against HIV, H1N1 influenza, herpes simplex virus-1, Coxsackie virus A9, or tick-borne encephalitis virus [82,83,84,85,86,87,88,89,90]. Although HAs have been reported to be without side effects over long-term use [33,35], there are indications that with long-term use, an acute deficiency of selenium will appear, which is probably complexed with HAs and eliminated from the body [64]. In agreement with this finding, HAs present in drinking water are indicated as a possible etiological factor of selenium deficiency, which is associated with the occurrence of endemic diseases and increased virulence of the coxsackie virus in some areas [91].

Cetin et al. [92] presented HAs as a suitable supportive supplement for managing social stress. It was also worthwhile monitoring the parameters of antioxidant defense in central metabolically active organs as well as in circulation (liver, kidney mitochondria, and plasma) when assessing the stress from animal transport on the redox state of the organism. According to the activities of antioxidant enzymes, the administration of HAs at 0.6% in feed mixtures over 42 days was considered to be beneficial for lowering stress, since oxidative stress conditions have not been demonstrated in the main organs responsible for nutrient metabolism and energy production [93].

It is also possible to include photoprotective effects here. Interesting photoprotective properties of HAs described by e.g., Muela et al. [94], studied on the bacterium *Escherichia coli* in an aqueous environment may be related to certain structural analogies with melanins (also above mentioned in precursors of HAs), especially the long, conjugated system of π-bonds (e.g., aromatic nuclei). Their electrons can be relatively easily excited by UV radiation, which means that this radiation is absorbed by such molecules and thus the risk of damage to tissues exposed to UV radiation is reduced. However, HS show effects by other mechanisms too. Reduced production or complete lack of melanin may lead to facial and dermatological problems in humans [95]. On the other hand, elevated synthesis of melanin is obvious in several skin disorders, including melasma, periorbital hyperpigmentation, lentigo senilis, and skin cancer risk [96]. Melanogenesis is a complicated process with numerous chemical and enzyme-catalyzed reactions. The most important role in melanin synthesis is played by tyrosinase (TYR) and tyrosine-related proteins (TYRP1 and TYRP2). TYR is a bifunctional and glycosylated copper-containing metalloenzyme with dinuclear copper ions widely distributed in prokaryotes, plants, and mammals [97]. Despite the well-known catalytic activity of tyrosinase, it is the only melanin-forming enzyme in humans [98,99]. The study of Taherkhani et al. [97] followed the inhibitory effects of FAs and HAs acids against TYR. Detection of the kinetic parameters for TYR after the addition of HAs and FAs into mixtures of tyrosine and L-DOPA unveiled a dose-dependent inhibitory action of humic compounds against the enzyme. The values of the Michaelis constant and maximal velocity signifies a mixed-type inhibition of TYR in the presence of HAs and FAs. The intrinsic fluorescence of TYR diminished regularly with increasing concentrations of HS, indicating variations in the tertiary structure of tyrosinase. There has been found a considerable effect of FAs on the secondary structure of TYR, while HAs could not affect it. The inhibitory activity of HAs and FAs can therefore be evaluated in the production of cosmetic products and in the medical, bioprocessing, agriculture, and environmental industry. Secondly, administration of HS attained a decrease in serotonin and its metabolite, 5-hydroxy indole acetic acid, while normalizing dopamine, and noradrenaline in rat’s brain [100]. Abnormal synthesis of (neuro)melanin in the human brain, from the excess of dopamine not accumulated in vesicles by a deficiency in vesicular monoamine transporter-2 is associated with Parkinson’s disease. Dopamine outside the vesicles, at a pH of around 7 (in the cytoplasm, extracellular space, and in the synaptic gap) undergoes autoxidation to cyclic aminochromes, which polymerize to melanin. Autooxidation is associated with the production of ROS and the toxic consequences of their production, directly in dopaminergic neurons but also in surrounding neural cells [101,102].

### 3.2. Adsorption Properties of Humic Acids

HAs also have the ability to adsorb a number of more or less toxic organic molecules, including pesticides, polycyclic aromatic hydrocarbons (PAHs), toxins, pharmaceuticals, etc., thus adsorbed HAs deactivate them to a certain extent and participate in their excretion from the body [103]. HAs, especially in their polymeric, water-insoluble form, have a significant potential for the adsorption of various inorganic, organic, low-molecular, or polymeric substances, and biopolymers. HAs are able to adsorb, for example, pesticides [104], polyaromatic hydrocarbons [105], mycotoxins, DNA [106], proteins (e.g., encapsulation of lysozyme, trypsin, and ribonuclease A enzymes [107]), herbicides, insecticides, and fungicides [108]. While the adsorption of substances of organic origin takes place mainly through relatively weaker dipole–dipole interactions (i.e., hydrogen bonds), hydrophobic interactions (so-called instant dipole reactions), or through the formation of aromatic charge-transfer complexes (so-called charge-transfer complexes), the adsorption of metal cations takes place on the contrary, either through relatively stronger ionic bonds, which are mainly used in the adsorption of cations with a small radius and high charge density, i.e., mainly s^1^ and s^2^ elements, or through the formation of variously stable coordination bonds with cations of p, d, and f metal elements. These differences implicitly result in the variability of the adsorption characteristics for individual types of substances, especially depending on the pH of the reaction environment. Similarly, although, in general, HAs can be considered a good adsorption material, it is also necessary to take into account the high structural diversity of HAs from different sources, which is also reflected in their different sorption properties towards different substances. Studies have shown that their interactions vary depending on their origin, molecular weight, and HA concentration [109]. Therefore, it is not expedient to assess HAs as a sorption material in general but to approach individual samples or commercial preparations individually when evaluating these properties. Thus, HAs appear as a potential remediating agent for the environment contaminated by a wide range of contaminants. They also represent a significant potential for the use of these chemical or physicochemical properties in the veterinary as well as human medicine.

#### 3.2.1. Adsorption of Metal Cations

Adsorption of metals is an important property of humic acids both from an environmental point of view, but also from the interesting point of view of the biological effects of HAs in the organism, where their interaction with metal cations can potentially be manifested on several levels, e.g., interaction with metalloenzymes or elimination of toxic metals in acute or chronic poisoning. Violation of the homeostasis of biologically significant metallic or semi-metallic elements (Fe, Cu, Zn, Se, etc.) can lead to a whole range of disorders or diseases, consisting in the inhibition of metalloenzymes or selenoenzymes, or an increased level of oxidative stress, either due to the promotion of the formation of reactive oxygen particles mainly in terms of the Fenton reaction or as a result of the indirect promotion of peroxidation reactions due to the inhibition of antioxidant enzymes (the latter is rather characteristic of heavy metals with low values of electrochemical redox potentials, which are not “Fenton active”, such as Pb, Hg). Two mechanisms of binding of heavy metals by HAs are described—the formation of a covalent bond, and the formation of a coordination bond when the metal accepts an electron pair from the ligand [110]. According to Zhou et al. [111] Pb, Fe complexation proceeded for approximately 20 s, and that of Cu held as long as 150 s, with C-O, N- and S- groups of HA playing an active role.

In vitro experiments have demonstrated the striking ability of HAs to chelate a wide group of metal ions. Martyniuk and Wieçkowska [112] compared the absorption capacities of two different samples of HAs extracted from lignite originating at two different mining regions of Poland, in powder form as well as in the form of a hydrated gel obtained as a product of incomplete purification of isolated HAs. The difference in ion selectivity between these four preparations was not striking, however, sorbents in the form of a gel generally showed only slightly higher selectivity towards heavy metals (Pb, Ag, Cd, Cu, Ba, Fe), but towards light metals (Mg, Al, Cr) up to approximately twice, three to four times higher selectivity to Fe. For monometallic solutions (with a concentration of 0.2 mol.l^−1^), the order of metals in terms of affinity to sorbents was as follows: Pb > Ag~Hg > Cd~Ba~Cu > Ni~Co~Mn~Zn~Ca > V~Fe~Mg~Al~Cr, while the affinities of Fe (II and III), Mg, Al and Cr to the gels were several times higher than to the powder sorbents, ferric and ferrous ions reached even higher values than copper ions. In multimetallic solutions imitating industrial wastewater, high selectivity towards lead ions was maintained, however, in the case of gel sorbents, affinities towards Cr^3+^ and Al^3+^ ions increased significantly, at the expense of Cd^2+^, Ni^2+^, Mn^2+^, Ca^2+^, Mg^2+^ ions. The analysis of IR spectra of isolated HA–metal complexes led to the conclusion that the main contributors to metal chelation are the carboxyl groups of humic acids, where at low pH (<5) only a part of these functional groups participate in active complexation, while at higher pH (~7) almost all of them are already included. However, Pb^2+^, Ag^2+^, Hg^2+^, and Cu^2+^ ions are also capable of complexing with phenolic −OH groups even in slightly acidic solutions (pH < 5), while the participation of these groups in the chelation of the other studied ions is generally negligible. It was also proven that all investigated metal ions, except Ag+, formed hydrocomplexes with HAs, while the complexes of Mg^2+^, Ni^2+^, and Al^3+^ ions were the most hydrated. Janoš et al. [113] evaluated the ability of commercially available oxyhumolite and iron humate (IH) sorbents to remove hexavalent chromium from aqueous solutions. The removal mechanism of this ion is two-step: in the first step, Cr^6+^ is reduced to Cr^3+^, which is subsequently chelated by the carboxyl groups of humic acids. In the case of HAs (oxyhumolite) itself, the reduction is ensured mainly by phenolic –OH groups, in the case of IH, the majority of the Fe^2+^ ion participates in the reduction, which is simultaneously oxidized to Fe^3+^. In the case of oxyhumolite, the ability to reduce Cr^6+^ to Cr^3+^ decreased with increasing pH, while it was no longer observed above pH~5. However, the sorption capacity towards total chromium increased on the contrary. At pH < 2, there was a reverse liberation of reduced chromic ions back into the aqueous phase. Although the adsorption capacity and reduction ability of IH was sufficient in a wider range of pH, at pH values above 5 the adsorption capacity was reduced, probably due to the coprecipitation of Fe^3+^ and Cr^3+^ hydroxides. An interesting finding was the fact that the use of phosphate buffer solutions reduced the adsorption capacity, that is, the anions of this buffer actively compete for the interaction with the functional groups of the sorbent. Klučáková and Pavlíková [114] investigated the adsorption of Cu, Pb, Zn, and Cd ions from their diluted solutions (0.003–1000 mg·L^−1^) onto a sorbent obtained by isolation from lignite with a high proportion of aromatic regions in its structure. The average percentage rate of adsorption from the solution of copper ions was 99.9% or from the mixed solution of all four ions was as follows: Cd^2+^ 90.3%, Cu^2+^ 94.4%, Pb^2+^ 92.8% and Zn^2+^ 92.0%, while depending on the initial concentration of the ions it fluctuated in the range of 80–100%, without obvious tendencies. However, the average percentage of total adsorption was in the order Pb^2+^ 31.7% > Cu^2+^ 23.7%~Zn^2+^ 23.6% > Cd^2+^ 21.0%. Analysis of back extraction into the aqueous phase or into a solution of 1 mol·L^−1^ HCl led to the conclusion that the HAs-Pb and HAs-Cu complexes have high stability (the percentage rate of reverse extractability did not exceed 20% even for the HCl solution), while the complexes with the remaining two metals were more labile (for cadmium up to over 25% for acid extraction). Overall, the results show a very good adsorptivity of the evaluated co-cations towards HAs of lignite origin. While strong chelating interactions of carboxyl groups contribute to the interaction of HAs–metals, the adsorption of metal ions from mixed solutions is accompanied by ion-exchange competition.

It was proved that HAs are absorbed through the skin [115]. Although, there is no direct evidence between the absorption of HAs and their amount in the gastrointestinal tract (GIT). HAs are at least partially absorbed in the GIT of rats and can subsequently be metabolized in the body. In practice, only a few studies on the effect of HAs as well as FAs on absorption in the GIT and the subsequent influence of microelement concentrations in other organs of animals have been performed [116]. Mezes et al. [117] showed that complexes of metals with organic chelating agents, such as HAs, increase the bioavailability of these metals. This property is a consequence of the structure of organometallic complexes, which protects and stabilizes trace elements during the passage through the GIT, thereby enabling or facilitating their flow through the intestinal wall via amino acid transport systems and thus, ensures a higher rate of passive diffusion due to reduced interaction with other nutrients. The effect was followed at different time intervals of HS administration. After 21 days of administration of HAs in the form of sodium humate, a decrease in the level of selenium in serum and plasma was detected in weaned piglets [118]. Additionally, Mn levels were decreased. Antagonist interactions between Mn and elevated Fe serum concentration potentially play a role here [119]. The increase in Fe was even more pronounced after 6 weeks when HAs were administered in rabbits [120]. However, HAs contribute to the maintenance of Fe homeostasis in the organism. As found by Szabo et al. [121] in the liver and kidney of rats, Fe concentrations were reduced, probably due to HAs strong free binding capacity and thus tighter binding of Fe. After 26 days of administration of HAs to rats, Hullár et al. [116] found that they have a significant dose-dependent stimulating effect on the absorption of Cu and Zn in the intestine. The HAs supplementation had no significant effect on Cu concentrations in the kidney, femur, and hair. In contrast, Zn content in the femur showed a tendency to decrease. Although the high binding ability of HAs to bivalent cations improves the absorption of elements, after 42 days of administration of HAs to chickens, this ability of HAs was not confirmed [93]. They are not even consistent with the findings of Herzig et al. [122]. However, this phenomenon in the administration of HA is partially confirmed by the findings of Islam et al. [123], who also proclaimed a decrease in Cu and Zn levels, which, returned to their original values after 60 days. Increased levels of Cu and Zn were also detected in the breast muscle of turkeys after 10 weeks of HA administration [124]. Reduced Mn concentrations in kidney and liver mitochondria but increased Se in the kidney were found after 42-day administration of 0.6% HA in chickens [63]. An increased Zn, Mn, Fe, and Se in liver and kidney mitochondria in chickens fed with 0.6% HAs for 42 days were detected also [92]. This is in accordance with the findings of Ipek et al. [125] and the described properties of HAs, as well as the antagonist effect between Fe and Cu or Zn anticipated by Creech et al. [119] and Žatko et al. [64].

In the study of Vašková et al. [93], a certain recess has been detected in the content of measured elements in chickens’ tissues. After the stressor affection from the transportation of the chickens, the concentrations of the Mn and especially the Cu (Zn only in the kidney mitochondria) were higher in the HAs-treated group compared to the control group of stress-free chickens. The only strong correlation was the activity of SOD depending on the concentration of Cu^2+^ ions in the organs of the control and experimental groups after transport [93]. However, in the group affected by stress, the Fe concentrations were generally lower. Thus, administration of HAs for 42 days was considered beneficial for coping with the stress of animals from transport, since oxidative stress conditions either from antioxidant parameter measurements in the main organs responsible for the metabolism of nutrients and energy production, or due to redistribution of metals based on unexpected requirements of the organism have not been proven [93].

In the case of intoxication, the ability of HAs to form complexes with heavy metals is very important to prevent their action in the body [126]. In cases of acute lead intoxication and chronic (Pb, Cu) poisoning, HS application in the form of a suspension can be very important because they are unable to pass the stomach walls and enter the bloodstream, as opposed to most conventional antidotes [127]. Humifulvate (HFC), a mixture of humic, fulvic, and phenol acids, extracted from peat in the Hungarian Lake Balaton, is used as a supplement for human use in the US. In 2003, it was approved by the FDA on the basis of documented clinical trials conducted in Hungary on a group of volunteers. According to these studies, the use of 313.4 mg/day of HFC (150 mg HAs) for six weeks has led to an increase in the concentration of Cu and Fe in blood, as well as the adjustment of iron metabolism. Furthermore, it was confirmed that doses 156.7 to 313.4 mg HFC/day = 75–150 mg/day HAs) have a therapeutic effect in heavy metal (such as Cd and Pb) poisoning. The doses mentioned for 3–12 weeks contributed to increased urinary excretion of cadmium and an overall significant reduction in the concentration of these toxic elements in the blood serum compared to the control group extra, effectively preventing Cd and Pb from dietary sources [128]. These results also point to the very significant ability of HAs in living systems to actively interact with ions of different metals. Therefore, they can serve as carriers for elements important for metabolism (Cu, Fe), albeit on the other hand, as a toxic elements remover, with which it tends to form highly-stable complexes. In accordance with studies, humic substances show interesting use in the case of acute Al(III) or Pb(II) poisoning, creating a complex with these metals and allowing their elimination from the body [129]. The stability of the resulting complexes may vary. Kostic et al. [130] found the greatest stability between Pb (II) and HAs and lowering with other metal ions in the order Co(II) < Cu(II) > Zn(II).

Authorization of HAs for the treatment of lead poisoning by the European Agency for the Evaluation of Medicinal Products [1] allowed testing of the properties of HAs in the elimination of chronic lead poisoning in an experiment with a total duration of 10 weeks. Increased concentrations of Zn and Se have been detected in some tissues after five weeks of chronic Pb intoxication and administration of two HAs concentrations, 0.5 and 1% at the same time in rats. As stated, for example, in the Roney and Colman study [131], in intoxicated asymptomatic workers with high Pb levels in the blood, Zn-induced metallothionein sequestering of Pb was detected. A significant increase in Se concentration in the liver in rats receiving 0.5% HAs was detected, probably as a result of the antagonistic effect of Se to the toxic effects of heavy metals [132], indicating the activation of other defense mechanisms. Increased Se concentrations have been detected in intoxication by metals with the explanation of selenide complexes formation allowing further elimination by the kidney [133]. Finally, when comparing the activities of antioxidant enzymes, in which active centers are also determined elements and maintenance of the reduced glutathione in all monitored organs, a beneficial therapeutic dose seemed to be 1% HAs [134]. Even the distribution of elements in bones, and on lower subcellular levels, in mitochondria of monitored organs, was mostly restored to the level of unintoxicated animals in a group with 1% HAs [135]. The studies monitored the concentrations of Pb, Se, Mn, Cu, Fe, Zn, and the activities of superoxide dismutase, glutathione peroxidase, glutathione reductase and reduced glutathione concentrations in liver, kidney, heart, bones, plasma and isolated heart, liver and kidney mitochondria (Table 1).

In connection with the ability of HAs to adsorb metal cations, the risk associated with e.g., their inhalation, Ghio and Madden [136] showed that inhalation of HS and humic-like substances (HULIS) from the air leads to the activation of inflammatory pathways and subsequent lung fibrosis. According to their results, intake (inhalation) of substances containing in their structure a whole range of groups (e.g., alcohol, diol, epoxy, ether, aldehyde/ketone, ester, phenol, catechol, etc.) leads to their deprotonation at physiological pH. Subsequently, cellular cations, preferentially iron, are complexed by these compounds, leading to functional iron deficiency within the cell. A cell is prone to oxidative stress, cell communication is affected, transcription factories are activated and pro-inflammatory mediators leading to apoptosis are released. Miners, but also people performing agriculture activities are exposed to an increased risk of inhaling HS and HULIS, which is considered a risk factor for the development of sarcoidosis [136,137,138].

#### 3.2.2. Adsorption of Mycotoxins

Mycotoxins are secondary metabolites produced by fungi and according to different structures divided into polycetoacids, terpenes, cyclopeptides, and nitrogen compounds. They are mainly produced by *Aspergillus*, *Penicillium*, *Fusarium*, *Claviceps,* and *Alternariagenera* spp. A respective kind of fungi is generally able to produce a variety of mycotoxins, one and the same toxin can be produced by several types of fungi. From an epidemiological point of view, however, the term mycotoxins is encountered especially in fungi products that attack agricultural crops during growing, by further handling and processing, they get to the food chain, and are capable of causing disease and death in animals as well as in humans after intoxication [139,140]. It is believed that the importance of synthesis for the parasitic fungi is to weaken the host, which ultimately allows better proliferation of their colonies. The toxicity of specific fungal mycotoxins varies considerably for various animal species, but their common feature is their high resistance to physical, chemical, and biological effects, resulting in generally high levels of fungal persistence in the environment, respectively, in the food chain [141]. Merely 40 from 300 mycotoxins are monitored [142], which barely captures the real health threat additionally multiplied by additivity, synergism, or antagonism of effects [143].

Kabak et al. [144] dealt with mycotoxin removal strategies at the process level of the food and feed industry. As one of the prospective alternatives, they described adsorption on polymer sorbent, which from the point of view of the mentioned industry should meet some important basic assumptions, that include e.g., high capacity for the adsorption of the greatest possible amount of mycotoxins, high affinity for the adsorption of very low concentrations of mycotoxins, low toxicity and level of burden on the environment, further stability of the sorbent in a wide range of pH, elucidated mechanism of sorbent–toxin interactions, and in vivo confirmed effectiveness against all major occurring mycotoxins. In practice, sorbents based on montmorillonite and bentonite are used. However, due to their properties, and various binding sites, HAs represent another alternative for the reduction in absorption and systemic accessibility of bacterial toxins [145,146].

A derivative of HAs, oxihumate, has been shown to be an effective adsorbent of aflatoxins in in vivo studies on chickens [102]. The adsorption ability of HAs and mineral clay for zearalenon (ZEA) and aflatoxin B1 (AFB_1_) was shown to be pH-dependent [147,148]. Evaluation of the binding capacity of oxihumate by Langmuir and Freundlich adsorption isotherms showed absorption up to 10.3, 7.4, and 11.9 mg of AFB_1_/g of oxihumate at pH 3, 5, and 7 in vitro [102]. HAs as feeding additives in a range of doses from 0.2% to 0.4% (*w*/*w*) improved biochemical parameters and mitigated organ damage [149]. Arafat et al. [150] found that the administration of HAs’ preparation in concentrations of 0.1%, 0.2% and 0.3% to broiler AFB_1_ intoxicated chickens led to a general alleviation of the symptoms of poisoning, consolidation of the total health status of chickens, maintenance of normal body weight, reduction in mortality, mitigation of marked increase in liver weight, and improved biochemical parameters in a dose-dependent manner. In vitro testing revealed decreasing bonding of HAs to aflatoxin with increasing aflatoxin concentration. Still, 100 mg/mL of HAs with the addition of 20 ppb aflatoxin showed an ability to bind up to 90.5%. In vivo experiments proved dose-dependent favorable effects against toxicity when examined in lipid and antioxidant parameters [151]. Recently, this possibility was verified by using vermicompost, whose content of HAs differs from other sources (lignite, leonardite, etc.) as it represents a substance still in the process of humification, but with proven biological properties in vivo [152,153,154]. Even 97.6% and 99.7% adsorption capacity for AFB_1_ were found for HAs and sodium-free HAs isolated from vermicompost in in vitro study [155]. Based on the detected effects and already mentioned properties of HAs, several mechanisms by which HAs bind aflatoxins have been proposed. They mainly include the mentioned electrostatic interactions, hydrogen bonds, π–π stacking [156], hydrophobic interactions [157], and the presence of aromatic structures and oxygen-containing functional groups [158].

The ability to effectively bind HAs and thus increase excretion from the body was also found for other toxins. Santos et al. [159] investigated the in vitro dependence of the adsorption of ochratoxin A (OTA) and ZEA on the applied concentration of HAs at different pH, imitating different environments of the gastrointestinal tract of monogastric animals (3.0-stomach, 7.4-oral cavity, 8.4-intestine). There was a generally directly proportional dependence of the percentage of adsorption on the concentration of HAs for both selected mycotoxins, however, in the case of OTA, adsorption was clearly most pronounced in an acidic environment, where this dibasic acid (pKa 4.4 and 7.3) was in an undissociated state, as well as the HAs matrix (possibility of forming hydrogen bonds with each other), but after deprotonation at a pH in the environment or above the second pKa, OTA already carried a negative charge, as a result of which it obviously lost the ability to form sufficiently strong physical or chemical attractive interactions (hydrogen bonds, non-polar interactions). This effect was hardly noticeable in the case of ZEA, which appeared as a monocytic acid with a pKa of 7.62, and thus more massive deprotonation occurs only in the case of the most basic conditions. Results of experiments with a constant concentration of HAs (4.0 mg·mL^−1^) and different initial concentrations of mycotoxins were correlated with several adsorption isotherms (Langmuir, Freundlich, Brunauer–Emmett–Teller Redlich–Peterson), and led to the finding that the adsorption of these toxins on HAs is basically monomolecular adsorption on energetically unequal adsorption centers. In in vitro tests at different pH imitating the gastric and intestinal conditions of the digestive tract of pigs, Sabater-Villar et al. [160] focused on the absorption properties of several sorbents, including six different samples of humic acids, against two specific mycotoxins: ZEA and deoxynivalenol (DON) (at a concentration of 1 mg·L^−1^). Except for activated carbon, none of the tested formulations showed the ability to efficiently bind DON (the maximum rate for one of the lignosulfonates was 21% at pH 8.0). Against ZEA, however, along with one of the clays and one of the yeast preparations, three of the six HAs samples showed a significantly high adsorption capacity. The ability of HAs to bind ZEA for these three samples increased from approximately 47% at the lowest to almost 100% at the highest of the applied concentrations, while in the alkaline environment, these values were only a few percent lower compared to the acidic conditions. From the analysis of the properties of the individual samples, it follows that the lower hydration capacity and the higher density of the respective sample of humic acids contribute to the higher adsorption capacity of ZEA. The difference in the adsorption capacity of ZEA and DON results from their different chemical structure. While ZEA is a polar macrolactone of resorcylic acid, DON is a tetracyclic epoxy-enone with two alcohol hydroxyl groups, thus it has considerably less polarity, the ability to form hydrogen bonds, and the ability to ionize in an alkaline environment. It is therefore obvious that it is not possible to unify the adsorption properties of HAs against the whole group by the effects of origin-related substances, but rather to investigate the effect of a specific sample of HAs with defined properties against a specific compound.

Previous studies showed DON to have significant potential to induce oxidative stress conditions, lipid peroxidation, and insufficiency in the activities of antioxidant enzymes due to the metabolism of DON [161,162,163,164]. In a 35-day experiment on rats assessing the antioxidant status of the liver, heart, kidney, and plasma, DON demonstrably caused the induction of oxidative stress, the intensity of which appears to be directly dependent on the concentration of the mycotoxin dose [165]. The activities of superoxide dismutase, glutathione peroxidase, glutathione reductase, glutathione-S-transferase and reduced glutathione concentrations in plasma, and isolated heart, liver and kidney mitochondria were detected (Table 2). HA supplementation led to changes in glutathione-related enzyme activities in the monitored organs, lowering stress conditions. The effect of HAs could be partly from adsorbing DON from the GIT and reducing its bioavailability and partly by actively interfering with the inhibition of oxidative stress conditions and/or affecting the redistribution and bioavailability of elements that are cofactors of antioxidant enzymes. The results indicate significant involvement of the glutathione-S-transferase-glutathione conjugation system.

## 4. Conclusion

The experience of using humic acids in the prevention and treatment of various diseases, as well as uncovering the mechanism of action in the body, confirm that these natural substances are very promising in the protection and promotion of a healthy state.

## Figures and Tables

**Table 1:** Design of the study of humic acids’ efficiency in chronic lead intoxication [134,135]. Study was provided on 210 Sprague–Dawley rats. Lead was administered at a daily dose of 155.5 mg/kg body weight in water. Approximately one-third of the animals in each group were analyzed on days 7, 35, and 70 of the experiment. Lead was administered only up to 35 days. From day 35 until the end of the experiment, the effectiveness of humic acids to reverse intoxication was monitored exclusively.

Day 0	Day 7	Day 35		Day 70
control				
Pb				
0.5% HAs	liver	liver	HAs dosage	liver
1% HAs	kidney	kidney	remain	kidney
2% HAs	heart	heart	Pb removed	heart
Pb + 0.5% HAs	plasma	plasma		plasma
Pb + 1% HAs	bones	bones		bones
Pb + 2% HAs				

**Table 2:** Design of the study of humic acids’ efficiency in deoxynivalenol intoxication [165]. In compliance with the 3R principles for animal experiments, dosage of 1% humic acids was intended according to previous studies [134,135]. Deoxynivalenol was administered *per os* (0.07% solution in 14% solution of sucrose) in the volume calculated for the current weight of the rats once a day.

72 Sprague-Dawley rats	control	35 days	
	1% HAs		liver
	DON exceeding limit in 100%		kidney
	DON excceding limit in 200%		heart
	DON exceeding limit in 100% + 1% HAs		plasma
	DON exceeding limit in 200% + 1% HAs		

## Data Availability

No new data were created or analyzed in this study. Data sharing is not applicable to this article.
